# Lifetime Ultraviolet Radiation Exposure and DNA Methylation in Blood Leukocytes: The Norwegian Women and Cancer Study

**DOI:** 10.1038/s41598-020-61430-3

**Published:** 2020-03-11

**Authors:** Christian M. Page, Vera Djordjilović, Therese H. Nøst, Reza Ghiasvand, Torkjel M. Sandanger, Arnoldo Frigessi, Magne Thoresen, Marit B. Veierød

**Affiliations:** 1 Oslo Centre for Biostatistics and Epidemiology, Oslo University Hospital, Oslo, Norway; 2 Centre for Fertility and Health, Norwegian Institute of Public health, Oslo, Norway; 3 Oslo Centre for Biostatistics and Epidemiology, Department of Biostatistics, Institute of Basic Medical Sciences, University of Oslo, Oslo, Norway; 4 Department of Community Medicine, UiT - the Arctic University of Norway, Tromsø, Norway; 5 Department of Research, Cancer Registry of Norway, Oslo, Norway

**Keywords:** DNA methylation, Predictive markers

## Abstract

Ultraviolet radiation (UVR) exposure is a leading cause of skin cancers and an ubiquitous environmental exposure. However, the molecular mechanisms relating UVR exposure to melanoma is not fully understood. We aimed to investigate if lifetime UVR exposure could be robustly associated to DNA methylation (DNAm). We assessed DNAm in whole blood in three data sets (n = 183, 191, and 125) from the Norwegian Woman and Cancer cohort, using Illumina platforms. We studied genome-wide DNAm, targeted analyses of CpG sites indicated in the literature, global methylation, and accelerated aging. Lifetime history of UVR exposure (residential ambient UVR, sunburns, sunbathing vacations and indoor tanning) was collected by questionnaires. We used one data set for discovery and the other two for replication. One CpG site showed a genome-wide significant association to cumulative UVR exposure (cg01884057) (p_nominal_ = 3.96e-08), but was not replicated in any of the two replication sets (p_nominal_ ≥ 0.42). Two CpG sites (cg05860019, cg00033666) showed suggestive associations with the other UVR exposures. We performed extensive analyses of the association between long-term UVR exposure and DNAm. There was no indication of a robust effect of past UVR exposure on DNAm.

## Introduction

Solar radiation is the major source of human exposure to ultraviolet radiation (UVR)^1^, and the major risk factor for cutaneous melanoma and keratinocyte skin cancers^2,3^. Exposure to artificial UVR (indoor tanning) also increases skin cancer risk, and is classified as carcinogenic to humans^4^.

Identification of biomarkers indicating past exposures is important in the study of chronic diseases and their etiology. In epidemiological studies, DNA methylation has been a strong marker of environmental exposure^5,6^. Exposure to smoking, air pollution, and heavy metals have consistently been linked to epigenetic changes, mainly to DNA methylation^7,8^. UVR exposure has also been linked to DNA methylation, as UVR exposure has been demonstrated to change the epigenetic profile of the epidermis^9^. An assessment of ambient UVR exposure and DNA methylation in CD4^+^ T-cells in European American individuals^10^ demonstrated an epigenome-wide significant association for cg26930596 (*PRKCZ*), but failed to replicate in an independent sample. An Australian study found an association between UVR exposure and total LINE-1 hypomethylation^11^. LINE-1 has often been used as a marker of genomic integrity, and a loss of methylation in LINE-1 is associated with global hypomethylation and with structural instability of the genome.

Global hypomethylation has been associated with multiple cancers, including bladder, liver, breast, kidney, colon and melanoma^12^. With UVR exposure as the main risk factor for melanoma, it is of interest to investigate if UVR exposure can affect epigenetic profiles, and if DNA methylation mediates the association between UVR exposure and the risk of melanoma. Our aim was to assess the former, i.e., whether DNA methylation in blood leucocytes is associated with life history of UVR exposure. We used data from the Norwegian Women and Cancer (NOWAC) study, a population-based cohort, with information on lifetime UVR exposure, which has shown consistent associations with skin cancer^13–18^. We studied genome-wide DNA methylation as well as global methylation, including imputation of LINE-1 specific CpGs, in whole blood. UVR exposure is the main driver for skin photoaging, and we also examined if lifetime UVR exposure could result in an accelerated epigenetic age, estimated from DNA methylation in leucocytes^19^. Analyses were performed in the discovery set and two replication sets.

## Materials and Methods

### Study samples

The NOWAC cohort includes 172 000 women aged 30–70 years (born 1927–1965) when included in 1991–2006 from a nationwide random sample (response 54%)^20^. Host characteristics and lifetime UVR exposure were collected through questionnaires at baseline and every 4–6 years. Approximately 50 000 women in the NOWAC cohort donated blood samples and constitute the postgenome cohort^21^. The present paper includes controls from three data sets from the postgenome cohort, all cancer-free women at the time of blood sampling and selected as controls in case-control studies of melanoma (discovery set, n = 183 controls), breast cancer (replication set R_1_, n = 191 controls)^22^, and lung cancer (replication set R_2_, n = 125 controls)^5,23^. Matching factors were time since blood sampling and year of birth (1943–1947, 1948–1952, 1953–1957).

The women gave written informed consent to donate blood samples for biomarker analyses. We confirm that all methods employed in the study were performed in accordance with the relevant guidelines and regulations.

### UVR exposure

On the basis of ambient UVR hours at place of residence, ambient UVR is categorized as low (northern Norway), medium-low (central Norway), medium (southwestern Norway), and highest (southeastern Norway)^24,25^. In the baseline and follow-up questionnaires, participants reported history of severe sunburns (never, 1, 2–3, 4–5, ≥6 times per year), average number of weeks spent on sunbathing vacations per year (never, 1, 2–3, 4–6, ≥7 weeks) and average use of an indoor tanning device (never; rarely; 1, 2, or 3–4 times/month; >1 time/week) in childhood (≤9 years), adolescence (10–19 years), and adulthood (>19 years)^24^. The reported frequencies of indoor tanning, sunbathing vacations, and sunburns were transformed into equivalents of yearly sessions and multiplied by the length of each interval^16^. The participants were then classified into five categories; non-exposed and quartiles. To capture the tail of the distribution, the upper quartile was further divided into two equally sized groups (i.e. six categories in total). Cumulative UVR exposure was constructed by summarizing the categories (i.e. scores 0–5) for indoor tanning and sunbathing vacations^16^.

### Covariates

Participants reported education (≤10, 11–13, ≥14 years), smoking (never, former, current smoker), and hair color (dark brown/black, brown, blond/yellow, red); which is the best measure of skin sensitivity to UVR in the NOWAC cohort^13,15^.

### DNA methylation analyses

DNA was extracted at the HUNT Biobank, Levanger, Norway, and methylation arrays were analyzed at the Institute for Genomic Medicine, Torino, Italy. DNA was extracted from the blood samples using the QIAsymphony DNA Midi Kit (Qiagen, Crawley, UK), and 1000 ng (discovery) and 500 ng (R_1_ and R_2_) of DNA were converted with bi-sulfite (EZ-96 DNA Methylation-Gold™ Kit, Zymo Research, Orange, CA, USA) according to manufacturer’s instruction.

The samples for the discovery set were randomly placed on the plates, and randomly assigned to a row/column position, with equally many cases and controls on each column and plate. The Illumina Infinium MethylationEPIC BeadChips were hybridized according to the manufacturer’s protocol. All predicted cross-hybridizing probes (44 210)^26^, out-of-band probes (2843), and all probes with at least one CpG with detection p-value above 0.8 (5504 CpGs) were removed. This left 775 528 CpGs in samples from 183 controls. DNA methylation at LINE-1 CpGs were imputed in the discovery set using the R-package *REMP*^27^ and its default pipeline, without removing cross-hybridizing probes. We assessed only LINE-1 methylation and not other repetitive elements, since this is by far the most studied marker of association between UVR and DNA methylation.

For R_1_ and R_2_, the Illumina Infinium HumanMethylation450 BeadChips were hybridized according to the manufacturer’s protocol. Plate specific batch effects were corrected using ComBat^28,29^. After quality control that included removal of CpGs with >20% missing and non-specific CpGs, 416412 autosomal CpGs remained for R_1_ and 450890 for R_2_. Quality controls have been described in detail for R_1_^22^ and R_2_^5^.

All three data sets had background subtraction and control normalization performed with *minfi* to reduce background noise and dye bias^30^. Beta mixture quantile normalization^31^ using the *wateRmelon* R-package^32^ was performed for type I and type II probes in the three sets jointly. Cell type composition was estimated using the Houseman algorithm^33^ with a reference data set from Reinius *et al*.^34^. White blood cell composition estimates were obtained for CD4^+^ and CD8^+^ T-cells, NK cells, B cells, monocytes, granulocytes, and we estimated the granulocytes-to-leukocytes ratio.

### Statistical analysis

Correlations between the five UVR variables were estimated using Pearson’s correlation coefficient, *r*. Linear regression was used to study associations between UVR exposure variables and estimated fraction of each cell type component, as well as the lymphocyte to neutrophil ratio, adjusting for age at sampling, smoking status, time in freezer, and data set.

The methylation values were transformed from beta-values to M-values using a logit2 transform. Smoking results in a strong, well-known pattern in the DNA methylation and as a quality control, we performed linear regression with smoking status as the main exposure and DNA methylation as the outcome.

In the genome-wide analysis, DNA methylation was modelled as the outcome and UVR as the covariate in a linear regression model for each CpG, adjusting for age, smoking status, and time in freezer. Additional adjustment was performed for hair color, as a marker of skin sensitivity^15^, and for cell type composition. We tested for interactions between cumulative UVR exposure and hair color in each CpG, and similarly between lifetime sunburns and hair color.

We present estimated regression coefficients with standard errors (SE). Note that, as we are testing for trends through ordered categorical exposure variables, the estimated regression coefficients should be interpreted with caution. Furthermore, we used non-negative matrix factorization to summarize the UVR exposure variables and hair color, and to cluster the individuals into three exposure groups. Analysis of variance (ANOVA) was used to test for differences between these groups with regard to each CpG.

All p–value adjustments for multiple testing were done with the Benjamini-Hochberg false discovery rate (FDR) procedure. A CpG site was defined as significant if the FDR adjusted p-value was <0.05, and as replicated if the nominal p-value in any of the replication sets was <0.05. Replication was attempted for the 20 CpGs with lowest p-values in the discovery set.

We attempted replication of the previously reported association between ambient UVR exposure and cg26930596 in the *PRKCZ* gene^10^ in all three sets using linear regression.

We assessed global DNA methylation by two indicators: the average over all measured CpGs, and by imputing methylation at CpG sites in LINE-1. Average methylation levels were analyzed using linear regression, with UVR as the exposure and average methylation as the outcome, adjusting for age, smoking, and time in freezer. The association between UVR exposure and LINE-1 CpGs was modeled with two models, one at the level of individual CpGs with linear regression and one at the level of LINE-1 subfamilies using linear mixed models, with subfamilies as grouping factor, adjusting for age, smoking, and time in freezer for both models.

Biological age (*PhenoAge*) was estimated based on the 513 CpGs published by Levine *et al*.^19^, out of which 512 were available in the discovery set, 506 in R_1_ and 505 in R_2_. Age acceleration phenotype was defined as the difference between the chronological age and the estimated biological age^35^. Linear regression was used with age acceleration as the outcome and UVR as the exposure, adjusting for smoking and time in freezer. All analyses were performed using R software^36^.

### Ethics approval

The Medical Ethical Committees of North Norway has approved the NOWAC study and the storage of human biological material, as well as each sub-study used in this project.

## Results

Women in the discovery and replication sets were older than women invited to the postgenome cohort (Table 1). Furthermore, R_2_ was older, recruited earlier, and had shorter time in freezer, lower education, and more non-smokers compared to the discovery and R_1_ sets. UVR exposures in the three sets are presented in Table 2, and R_2_ had lowest proportion of women from the region with highest ambient UVR. Low correlation was found between residential ambient UVR and the other four UVR variables (−0.06 ≤ *r* ≤ 0.14), and between lifetime sunburns and the other UVR variables (0.09 ≤ *r* ≤ 0.16). Indoor tanning and sunbathing vacations were moderately correlated (*r* = 0.30).

**Table 1:** Characteristics of the women in the discovery and replication sets, and the women invited to participate in the postgenome cohort.

		Discovery set	Replication set R_1_	Replication set R_2_	Invited to the postgenome cohort
n		183	191	125	97474
Age, years	Mean (SD)	55.7 (4.2)	55.4 (4.4)	56.6 (4.0)	53.9 (4.2)
Birth cohort	1943–1947	73 (39.9)	76 (39.8)	67 (50.7)	31920 (32.7)
	1948–1952	70 (38.3)	62 (32.5)	41 (31.0)	33466 (34.3)
	1953–1957	40 (21.9)	53 (27.7)	24 (18.2)	32088 (32.9)
Recruitment year	1991–1992	81 (44.3)	78 (40.8)	70 (51.2)	35801 (36.7)
	1996–1997	29 (15.8)	42 (22)	28 (20.1)	13580 (13.9)
	2003–2007	73 (39.9)	71 (37.2)	32 (25.6)	48093 (49.3)
Time in freezer, years	Mean (SD)	11.21 (0.95)	10.37 (0.97)	9.16 (1.08)	NA
Education, years	≤10	54 (29.5)	62 (32.5)	48 (36.7)	29127 (29.9)
	11–13	55 (30.1)	53 (27.7)	42 (31.8)	29097 (29.8)
	≥14	67 (36.6)	68 (35.6)	31 (23.5)	34708 (35.6)
	Missing	7 (3.8)	8 (4.2)	11 (8.3)	4542 (4.7)
Smoking	Non-smoker	61 (33.3)	70 (36.6)	57 (39.5)	34339 (35.2)
	Former	73 (39.9)	61 (31.9)	36 (27.3)	36568 (37.5)
	Current	46 (25.1)	58 (30.4)	39 (29.5)	24289 (25.9)
	Missing	3 (1.6)	2 (1)	0 (0)	2278 (2.3)
Hair color	Black/dark brown	33 (18.0)	27 (14.1)	28 (21.2)	15202 (15.6)
	Brown	75 (41.0)	72 (37.7)	43 (32.6)	36783 (37.7)
	Blond/yellow	60 (32.8)	76 (39.8)	48 (36.4)	34089 (34.9)
	Red	5 (2.7)	9 (4.7)	3 (2.3)	3125 (3.2)
	Missing	10 (5.5)	7 (3.7)	10 (7.6)	8275 (8.5)

**Table 2:** Ultraviolet radiation (UVR) exposure in the discovery and the replication sets.

	Discovery set	Replication set R_1_	Replication set R_2_
n	183	191	125
**Residential ambient UVR**			
Low (northern Norway)	33 (18)	39 (20.4)	32 (25.6)
Medium-low (central Norway)	22 (12)	26 (13.6)	13 (10.3)
Medium (south-west Norway)	26 (14.2)	30 (15.7)	20 (15.9)
Highest (south-east Norway)	102 (55.7)	96 (50.3)	59 (47.4)
Missing	0	0	1 (0.8)
**Lifetime no. of sunburns**^**a**^			
0	24 (13.1)	25 (13.1)	20 (16.0)
1–20	42 (23)	60 (31.4)	32 (25.6)
21–30	24 (13.1)	26 (13.6)	18 (14.4)
31–47	45 (24.6)	41 (21.5)	21 (16.8)
48–58	21 (11.5)	17 (8.9)	18 (14.4)
59+	20 (10.9)	21 (11)	13 (10.4)
Missing	7 (3.8)	1 (0.5)	3 (2.3)
**Lifetime no. of sunbathing vacations**^**a**^			
0	14 (7.7)	11 (5.8)	7 (5.6)
1–29	44 (24)	40 (20.9)	25 (20.0)
30–62	44 (24)	56 (29.3)	35 (28.0)
63–104	41 (22.4)	36 (18.8)	31 (24.8)
105–143	16 (8.7)	18 (9.4)	13 (10.4)
144+	17 (9.3)	23 (12)	10 (8.0)
Missing	7 (3.8)	7 (3.7)	4 (3.2)
**Lifetime no. of indoor tanning sessions**^**a**^			
0	49 (26.8)	54 (28.3)	38 (31.2)
1–14	32 (17.5)	41 (21.5)	18 (14.4)
15–24	38 (20.8)	39 (20.4)	26 (20.8)
25–120	32 (17.5)	25 (13.1)	17 (13.6)
121–418	18 (9.8)	10 (5.2)	13 (10.4)
419+	14 (7.7)	22 (11.5)	12 (9.6)
**Cumulative UVR**^**b**^			
0	10 (5.5)	7 (3.7)	7 (5.6)
1	18 (9.8)	13 (6.8)	11 (8.8)
2	20 (10.9)	37 (19.4)	13 (10.4)
3	31 (16.9)	25 (13.1)	17 (13.6)
4	26 (14.2)	24 (12.6)	22 (17.6)
5	25 (13.7)	32 (16.8)	25 (20.0)
6	24 (13.1)	22 (11.5)	7 (5.6)
7	15 (8.2)	13 (6.8)	11 (8.8)
8	7 (3.8)	13 (6.8)	7 (5.6)
9	5 (2.7)	4 (2.1)	3 (2.4)
10	2 (1.1)	1 (0.5)	2 (1.6)

^a^Categorized in six categories; non-exposed and quartiles, with the upper quartile divided into two equally sized groups.

^b^Sunbathing vacations and indoor tanning.

When testing each cell type independently, the UVR exposure variables were not significantly associated with cell type composition in any of the three sets, (0.06 ≤ p_adjusted_ ≤ 0.98) (Supplementary Table S1). The lymphocyte to neutrophil ratio was also not significant for any UVR exposure (0.07 ≤ p_adjusted_ ≤ 1). A total of 326 758 CpGs were present in all three sets. In the analysis of smoking, 113 CpG sites had p_adjusted_ < 0.05, of which 58 were replicated in at least one of the two replication sets (Supplementary Table S2).

### Differentially methylated CpG sites

The top 20 CpGs associated with each UVR exposure are listed in Supplementary Table S3. Two of the top 20 CpGs replicated in either R_1_ (sunburns and cg00033666) or R_2_ (ambient UVR and cg05860019). One CpG (cg01884057) was genome-wide significantly associated with UVR exposure (cumulative UVR) in the discovery set (p_adjusted_  = 0.03), but it was not replicated neither in R_1_ (p_nomial_ = 0.64) nor in R_2_ (p_nomial_ = 0.42) (Table 3). After further adjustment for hair color, the CpG associated with lifetime sunburns (cg00033666) was replicated (Supplementary Table S4). One new CpG replicated in this model; for sunbathing vacations (cg19577365) (Supplementary Table S4).

**Table 3:** Differentially methylated CpGs in the discovery set, either genome-wide significant, or replicated in replication sets R_1_ or R_2_.

Exposure	Position	CpG	Gene	Relation to Gene	Discovery set, n = 183			Replication set R_1_, n = 191			Replication set R_2_, n = 125		
					Coeff.	SE	p_nominal_	Coeff.	SE	p_nominal_	Coeff.	SE	p_nominal_
Residential ambient UVR	chr15: 53095195	cg05860019	*ONECUT1*^a^	Shore	−0.132	0.03	2.2e-05	0.0075	0.03	0.799	−0.075	0.03	**0.043**
Lifetime no. of sunburns	chr15: 96887043	cg00033666	*NR2F2*^a^	Shore	0.0699	0.01	4.69e-05	0.0346	0.01	**0.027**	0.00902	0.02	0.702
Cumulative UVR^b^	chr2:25150051	cg01884057	*ADCY3*^a^	OpenSea	0.1201	0.02	3.96e-08	−0.0083	0.01	0.639	0.01924	0.02	0.420

Adjusted for age, smoking, and time in freezer. Regression coefficients (Coeff.), standard errors (SE), and nominal p-value. The CpGs are annotated to their nearest gene. Replicating p-values are marked in bold.

^a^Closest annotated gene.

^b^Sunbathing vacations and indoor tanning.

We tested for interaction between lifetime sunburns and hair color and found no interaction for any of the CpGs (p_adjusted_ ≥ 0.42, discovery set). When testing the interaction between lifetime cumulative UVR and hair color, significant interaction was found for one CpG (cg15277477, p_adjusted_ = 4.1e-3), but this was not replicated (p_nominal_ = 0.81 in R_1_ and p_nominal_ = 0.99 in R_2_). After adjustment for cell type composition (Supplementary Table S3), no substantial differences were observed. The correlation coefficients between the top 20 effect estimates in the model with and without this adjustment ranged from 0.80 to 0.99.

The ANOVA comparing each CpG between the groups from the cluster analyses, identified two in the top 20 CpGs that were replicated: cg21452538 (p_nominal_ = 3.69e-5 in discovery) was replicated in R_1_ (p_nominal_ = 0.03) and cg05967123 (p_nominal_ = 2.75e-5 in discovery) in R_2_ (p_nominal_ = 0.02). The main driver of these associations was a factor composed of sunbathing vacations and cumulative UVR exposure.

### CpG site indicated in the literature

The CpG cg26930596 in the *PRKCZ* gene, previously reported to be associated with ambient UVR exposure, was significantly associated with ambient UVR exposure in R_1_ (p_nominal_ = 9.34e-3), but not in the discovery set (p_nominal_ = 0.65) or in R_2_ (p_nominal_ = 0.28).

### Global DNA methylation

Average methylation was not associated with any of the UVR exposure variables in the discovery or replication sets (0.06 ≤ p_nominal_ ≤ 0.93) (Supplementary Table S5). Indoor tanning and cumulative UVR exposure had negative effect estimates in all three sets, sunbathing vacation had positive effect estimates in all three sets, while lifetime sunburns and ambient UVR had a positive effect estimate in the discovery set and negative estimates in both replication sets. In the discovery set, no LINE-1 CpG was significantly associated with any of the UVR exposure variables (data not shown). No LINE-1 subfamily was significantly associated with any of the UVR exposure variables (Supplementary Table S6).

### Accelerated aging

Accelerated aging was associated with sunbathing vacations in R_2_ (regression coefficient = 1.8, SE = 0.48, p_nominal_ = 1.20e-3), but not in the other two sets (0.08 ≤ p_nominal_ ≤ 0.32). The remaining four UVR exposure variables were not significantly associated with accelerated aging (0.06 ≤ p_nominal_ ≤ 0.88; with the lowest p-value for cumulative UVR in R_2_).

## Discussion

We investigated the association between five UVR exposure variables (residential ambient UVR exposure, lifetime sunburns, lifetime sunbathing vacations, lifetime indoor tanning, and cumulative UVR exposure) and DNA methylation in lymphocytes in a discovery and two replication sets from the NOWAC cohort.

Only one CpG (cg01884057) site was associated with cumulative UVR exposure, but this finding was not replicated. Additionally, two CpGs were suggestively associated with the other four UVR exposure variables and replicated in one of the replication sets.

The CpG associated with cumulative UVR in our study lies in a DNase hypersensitive region 7 kb upstream of the Adenylate Cyclase 3 (*ADCY3*) gene, shown to be a potential oncogene^37^. However, no robust association with skin cancer has been indicated. Ambient UVR exposure was suggestively associated to a CpG (cg05860019) about 10 kb upstream, in the shore of a CpG Island associated to the One cut homeobox 1 (*ONECUT1*) gene. This gene is mainly transcribed in liver cells, but is important for cell cycle regulation and potentially associated with tumorigenesis or metastasis of malignant tumors^38^. The CpG suggestively associated with lifetime sunburns (cg00033666) lies in the shore of a CpG Island next to the master regulator gene Nuclear Receptor subfamily 2, group F (*NR2F2*). This gene has been suggested as an inhibitor target for melanoma and other cancers^39^. Somatic mutations in *NR2F2* have been observed in about 1% of melanomas^40^.

There are few studies on UVR exposure and DNA methylation, and most of the existing studies focus on cell lines or short-term exposure to UVR. The most similar study to ours in terms of design is the study by Aslibekyan *et al*.^10^, who investigated ambient UVR exposure and DNA methylation in CD4^+^ T-cells, which have been shown to express the CCR10 receptor when stimulated with sun induced vitamin D_3_^41^. One CpG from Aslibekyan *et al*.^10^ was nominally significant (cg26930596) in one of our samples, but with an effect estimate in the opposite direction.

The average beta-value across all methylation probes was not associated with any of the UVR exposure variables, but we observed an indication (not statistically significant) of hypomethylation. This is in line with previous research, which has observed a loss of DNA methylation after UVR exposure^11^. UVR exposure has been linked to LINE-1 hypomethylation in previous studies, but this has not been translated into an increased risk of melanoma^42^. LINE-1 methylation is often used as an indicator for global methylation. In this study, we used both average methylation over all observed CpGs, and imputed CpG levels at LINE-1. Neither was significantly associated with any of the UVR exposures.

UVR exposure is a primary driver of photo aging in the skin, and it can be hypothesized that other tissues could also show accelerated aging after UVR exposure. However, the association between sunbathing vacations and accelerated aging observed in R_2_ was not very strong, and was not found in the discovery or the R_1_ sets. No other UVR exposures showed a significant association.

An important strength of our study is the detailed life history of solar and artificial UVR exposure in the population-based NOWAC cohort, which has been consistently associated with risk of cutaneous melanoma^13,15–17^ and squamous cell carcinoma^14,18^. Indoor tanning irradiances are high in UVA radiation^43^ while UVB is the main cause of sunburns^44^. The intensity of the UVR exposure could not be directly assessed through the questionnaires, but since the UVR questions were segmented into age intervals in decades for each individual, estimates of dose were obtained.

An exposure with a demonstrated strongest epigenetic footprint, smoking, has been extensively studied, also in the NOWAC study^5^. As a quality control of the methylation data, we studied the associations with smoking in all three data sets. All significant probes that replicated across our sets have been previously reported in a large meta-analysis on methylation and smoking^45^ and demonstrate that the data were of sufficient quality and sample size to find biomarkers of strong exposures.

A weakness of our study is the lack of a directly exposed tissue, and the use of whole blood over skin samples. When studying the epigenetic patterns relating to environmental exposures or diseases, being as close as possible (in time and space) to the affected tissue is important since the epigenetic profile differs between tissues. Different cell types will also respond differently to the same environmental exposure. However, this has to be balanced against the availability of bio-samples. Large scale, general purpose biobanks, suitable for pre-diagnostic sampling will usually store only blood samples. While skin is the primary exposed tissue to UVR, and thus the most relevant tissue for studying direct effects of the UVR exposure, secondary effects of chronic UVR exposure might also be observed elsewhere, including in circulating lymphocytes^46^. The suppression of the immune system by UVR exposure is documented and is used as treatment for some autoimmune diseases^47^. Under the hypothesis that sustained UVR exposure influences the immune system, the cell type composition would be affected by the UVR exposure, and thus act as a mediator on the path from exposure to outcome. This places cell type composition on the causal pathway between UVR exposure and DNA methylation. Thus, after adjusting for cell type composition we will not be able to identify the total effect of the UVR exposure on DNA methylation, but rather the direct effect that is not caused by changes in the immune cell composition. We did not observe any strong associations between UVR exposures and the estimated white blood cell composition. The only UVR exposure variable showing some potential association with cell type composition was residential ambient UVR, which is also potentially confounded by population substructure. Given that no other UVR exposure showed a consistent association with cell type composition, this association is more likely caused by other factors than UVR exposure. Moreover, additional adjustment for cell type composition did not change the results.

The three data sets were collected as controls for case-control studies of melanoma (discovery), breast cancer (R_1_) and lung cancer (R_2_), which explains the older age of these sets compared to all women invited to the postgenome cohort and also the differences in time in freezer. The long-term storage of whole blood and DNA in biobanks may have a negative effect on DNA yield, but the integrity of DNA methylation does not seem to be affected by this^48^. However, there may be systematic differences between the three samples that are reflected in the time spent in freezer, such as difference in lab procedures, and this variable may serve as a proxy for such differences. To make the three samples more comparable, we therefore included this adjustment in all models.

UVR exposure is the main risk factor for skin cancer, but if this risk is mediated by DNA methylation is still not determined. We have made an extensive analysis of the potential association between UVR exposure and DNA methylation in blood, investigating the problem using different statistical approaches. Thus, we feel confident that long term UVR exposure has little effect on DNA methylation, and if DNA methylation is acting as a mediator of the melanoma risk from chronic UVR exposure, this is not reflected in DNA methylation in white blood cells.

## Supplementary information

Supplementary tables 1-6.

## Data Availability

The DNA methylation data generated and/or analyzed in the current study can be accessed upon reasonable request to the originating cohort. Access will be conditional on adherence to both local and national ethical and security policy. R codes used for the analyses presented in the paper are available upon request.
