# Supplementary material for: Lifetime Ultraviolet Radiation Exposure and DNA Methylation in Blood Leukocytes: The Norwegian Women and Cancer Study
Source: Sci Rep. 2020 Mar 11;10:4521. doi: 10.1038/s41598-020-61430-3 (PMC7066249; doi:10.1038/s41598-020-61430-3)
Supplement: Supplementary file 1 — Supplementary tables 1-6. [file 41598_2020_61430_MOESM1_ESM.pdf]

LIFETIME ULTRAVIOLET RADIATION EXPOSURE AND DNA METHYLATION IN BLOOD LEUKOCYTES: THE NORWEGIAN WOMEN AND CANCER STUDY

Christian M Page[1,2]

Vera Djordjilović[3]

Therese H Nøst[4]

Reza Ghiasvand[3,5]

Torkjel M Sandanger[4]

Arnoldo Frigessi[1,3]

Magne Thoresen[3]

Marit B Veierød[3,*]

Affiliations

[1]Oslo Centre for Biostatistics and Epidemiology, Oslo University Hospital, Oslo, Norway

[2]Centre for Fertility and Health, Norwegian Institute of Public health, Oslo, Norway

[3]Oslo Centre for Biostatistics and Epidemiology, Department of Biostatistics, Institute of Basic Medical Sciences, University of Oslo, Oslo, Norway

[4]Department of Community Medicine, UiT - the Arctic University of Norway, Tromsø, Norway

[5]Department of Research, Cancer Registry of Norway, Institute of Population-Based Cancer Research, Oslo, Norway

*Corresponding author: m.b.veierod@medisin.uio.no

Supplementary Table 1 Regression coefficients (Coeff.), standard errors (SE), and p-values (nominal and adjusted for the false discovery rate) for the association between ultraviolet radiation (UVR) exposure and estimated cell type proportions, adjusted for age, smoking, time in freezer, and data set (discovery, R_1, R_2).

	Coeff.	SE	p_{nomial}	$p_{adjusted}$
**Residential ambient UVR**				
**Monocytes**	0.00193	0.00101	0.05855899	0.08198259
**B**	-0.00183	0.00082	0.02746893	0.06409417
**CD4⁺ T**	0.00328	0.00337	0.33071205	0.38583072
**NK**	-0.00405	0.00179	0.02477869	0.06409417
**CD8⁺ T**	-0.00859	0.00375	0.02246377	0.06409417
**Gran**	0.00808	0.00390	0.03882304	0.06794032
**GLR[a]**	0.01498	0.03139	0.63341844	0.63341844
**Lifetime no. of sunburns**				
**Monocytes**	8.389e-4	0.00078	0.2868967	0.6694256
**B**	-7.451e-4	0.00064	0.2472909	0.6694256
**CD4⁺ T**	2.045e-4	0.00262	0.9379227	0.9833940
**NK**	-2.926e-5	0.00140	0.9833940	0.9833940
**CD8⁺ T**	5.692e-4	0.00293	0.8460938	0.9833940
**Gran**	-1.627e-3	0.00303	0.5919822	0.9833940
**GLR[a]**	3.542e-2	0.02405	0.1415681	0.6694256
**Lifetime no. of sunbathing vacations**				
**Monocytes**	0.00046	0.00089	0.6038581	0.9249528
**B**	-0.00037	0.00073	0.6097619	0.9249528
**CD4⁺ T**	0.00021	0.00297	0.9412630	0.9412630
**NK**	-0.00041	0.00159	0.7928167	0.9249528
**CD8⁺ T**	0.00139	0.00332	0.6742464	0.9249528
**Gran**	-0.00142	0.00344	0.6798482	0.9249528
**GLR[a]**	-0.03520	0.02765	0.2037118	0.9249528
**Lifetime no. of indoor tanning sessions**				
**Monocytes**	-0.00031	0.00074	0.67487881	0.9343641
**B**	0.00010	0.00060	0.86831136	0.9343641
**CD4⁺ T**	-0.00251	0.00246	0.30737230	0.9343641

**NK**	0.00222	0.00131	0.09182378	0.6427665
**CD8$^+$ T**	0.00028	0.00275	0.91896077	0.9343641
**Gran**	-0.00098	0.00285	0.73161563	0.9343641
**GLR[a]**	0.00189	0.02298	0.93436409	0.9343641
**Cumulative UVR[b]**				
**Monocytes**	-5.303e-5	0.00052	0.9200627	0.9200627
**B**	-1.558e-4	0.00043	0.7174105	0.9099034
**CD4$^+$ T**	-1.423e-3	0.00174	0.4153448	0.9099034
**NK**	1.291e-3	0.00093	0.1669923	0.9099034
**CD8$^+$ T**	5.456e-4	0.00195	0.7799172	0.9099034
**Gran**	-9.852e-4	0.00202	0.6267690	0.9099034
**GLR[a]**	-1.044e-2	0.01625	0.5209029	0.9099034

[a]Granulocyte to Lymphocyte ratio (proxy for Neutrophil to Lymphocyte ratio)
[b]Sunbathing vacations and indoor tanning

Supplementary Table 2 Regression coefficients (Coeff.), standard errors (SE), and adjusted p-values (false discovery rate; FDR) for smoking and DNA methylation in the discovery set. All genome wide significant CpGs on the FDR level of 0.05 were considered for replication. Replicated CpGs are marked in bold in the replication the R_1 and R_2 sets. CpGs not present on the 450k chip are marked as NA (not applicable).

	**Discovery set**			**Replication set R1**			**Replication set R2**		
	Coeff.	SE	$p_{adjusted}$	Coeff.	SE	$p_{adjusted}$	Coeff.	SE	$p_{adjusted}$
**cg05575921**	-1.14	0.080	1.558e-30	-0.86	0.067	**4.657e-27**	-0.85	0.083	**6.737e-18**
**cg21566642**	-0.49	0.039	1.806e-25	-0.53	0.046	**4.787e-23**	-0.55	0.051	**2.933e-19**
**cg01940273**	-0.39	0.034	3.926e-23	-0.32	0.033	**2.278e-18**	-0.36	0.037	**1.565e-16**
**cg03636183**	-0.41	0.041	6.025e-19	-0.39	0.037	**2.347e-20**	-0.39	0.045	**2.575e-14**
**cg17739917**	-0.42	0.044	1.274e-17	NA	NA	NA	NA	NA	NA
**cg21161138**	-0.30	0.033	2.890e-16	-0.26	0.029	**6.318e-16**	-0.30	0.041	**6.787e-11**
**cg14391737**	-0.45	0.052	3.800e-15	NA	NA	NA	NA	NA	NA
**cg18110140**	-0.32	0.040	9.449e-14	NA	NA	NA	NA	NA	NA
**cg19859270**	-0.43	0.053	1.421e-13	-0.23	0.036	**5.339e-10**	-0.15	0.052	**2.978e-3**
**cg17087741**	-0.54	0.068	2.925e-13	-0.15	0.080	**4.981e-2**	-0.13	0.093	1.534e-1
**cg03329539**	-0.21	0.028	1.891e-12	-0.20	0.029	**8.604e-11**	-0.23	0.039	**2.306e-8**
**cg16841366**	-0.30	0.041	7.792e-12	NA	NA	NA	NA	NA	NA
**cg02978227**	-0.30	0.043	2.732e-11	NA	NA	NA	NA	NA	NA
**cg21911711**	-0.40	0.057	6.511e-11	NA	NA	NA	NA	NA	NA
**cg26703534**	-0.23	0.033	7.419e-11	-0.16	0.025	**3.530e-10**	-0.14	0.031	**1.453e-5**
**cg19572487**	-0.30	0.044	7.725e-11	-0.21	0.035	**7.131e-9**	-0.21	0.045	**5.668e-6**
**cg06644428**	-0.63	0.093	1.706e-10	-0.46	0.075	**4.03e-9**	-0.55	0.107	**1.233e-6**
**cg25189904**	-0.31	0.047	5.405e-10	-0.31	0.045	**3.935e-11**	-0.33	0.062	**3.315e-7**
**cg25197654**	-0.19	0.030	7.061e-10	NA	NA	NA	NA	NA	NA
**cg04182427**	-0.32	0.049	8.286e-10	NA	NA	NA	NA	NA	NA
**cg15180656**	-0.25	0.040	2.352e-9	NA	NA	NA	NA	NA	NA
**cg04180924**	-0.22	0.035	3.099e-9	NA	NA	NA	NA	NA	NA
**cg13193840**	-0.25	0.040	3.278e-9	-0.15	0.049	**1.770e-3**	-0.25	0.061	**9.023e-5**
**cg26963277**	-0.36	0.058	4.003e-9	-0.22	0.052	**3.708e-5**	-0.27	0.099	**7.710e-3**
**cg11660018**	-0.20	0.032	4.723e-9	-0.14	0.027	**1.354e-7**	-0.15	0.037	**1.086e-4**
**cg25648203**	-0.30	0.050	6.478e-9	-0.20	0.030	**3.416e-10**	-0.23	0.045	**7.904e-7**

**cg05086879**	-0.43	0.071	6.610e-9	NA	NA	NA	NA	NA	NA
**cg15342087**	-0.32	0.053	1.066e-8	-0.14	0.022	**6.248e-10**	-0.15	0.034	**1.627e-5**
**cg25001882**	-0.17	0.030	1.594e-8	NA	NA	NA	NA	NA	NA
**cg13633560**	-0.17	0.029	1.748e-8	-0.09	0.030	**2.981e-3**	-0.13	0.044	**4.151e-3**
**cg00475490**	-0.40	0.068	1.754e-8	NA	NA	NA	NA	NA	NA
**cg10788371**	-0.17	0.029	1.872e-8	-0.09	0.027	**8.504e-4**	-0.15	0.031	**2.811e-6**
**cg06421013**	-0.23	0.039	2.138e-8	NA	NA	NA	NA	NA	NA
**cg27241845**	-0.21	0.036	2.296e-8	-0.15	0.026	**3.706e-8**	-0.16	0.035	**1.159e-5**
**cg07202214**	-0.16	0.028	2.537e-8	-0.09	0.030	**1.473e-3**	-0.14	0.041	**5.781e-4**
**cg24859433**	-0.38	0.065	2.739e-8	-0.18	0.026	**2.295e-11**	-0.15	0.033	**5.409e-6**
**cg26768182**	-0.36	0.062	2.760e-8	NA	NA	NA	NA	NA	NA
**cg11902777**	-0.33	0.058	3.576e-8	-0.19	0.038	**9.650e-7**	-0.25	0.054	**5.243e-6**
**cg20832643**	-0.40	0.070	3.658e-8	NA	NA	NA	NA	NA	NA
**cg01899089**	-0.13	0.023	4.136e-8	-0.07	0.020	**5.493e-4**	-0.10	0.034	**3.640e-3**
**cg02451831**	-0.32	0.057	5.237e-8	-0.09	0.022	**2.211e-5**	-0.12	0.032	**1.539e-4**
**cg09935388**	-0.53	0.094	5.598e-8	-0.24	0.060	**6.707e-5**	-0.39	0.080	**3.634e-6**
**cg10765427**	-0.11	0.020	5.883e-8	NA	NA	NA	NA	NA	NA
**cg14466441**	-0.22	0.039	9.132e-8	NA	NA	NA	NA	NA	NA
**cg23233742**	-0.15	0.028	1.051e-7	-0.05	0.025	**2.197e-2**	-0.14	0.027	**4.049e-7**
**cg25305703**	-0.32	0.059	1.312e-7	-0.14	0.040	**3.442e-4**	-0.19	0.054	**5.993e-4**
**cg15160624**	-0.27	0.050	1.348e-7	NA	NA	NA	NA	NA	NA
**cg20899321**	-0.24	0.044	1.368e-7	-0.006	0.032	8.333e-1	0.02	0.060	7.307e-1
**cg11931220**	-0.15	0.028	1.502e-7	NA	NA	NA	NA	NA	NA
**cg07741821**	-0.22	0.041	1.708e-7	NA	NA	NA	NA	NA	NA
**cg15187398**	-0.18	0.034	1.931e-7	-0.13	0.032	**5.874e-5**	-0.13	0.040	**1.655e-3**
**cg26361535**	-0.32	0.060	2.145e-7	-0.12	0.032	**1.261e-4**	-0.14	0.048	**4.027e-3**
**cg08064403**	-0.20	0.037	2.170e-7	NA	NA	NA	NA	NA	NA
**cg25949550**	-0.26	0.049	3.198e-7	-0.16	0.031	**2.284e-7**	-0.13	0.037	**4.416e-4**
**cg25845814**	-0.32	0.060	3.654e-7	NA	NA	NA	NA	NA	NA
**cg23771366**	-0.16	0.031	4.308e-7	-0.13	0.025	**2.298e-7**	-0.14	0.038	**1.895e-4**
**cg12803068**	0.78	0.152	6.120e-7	0.32	0.076	**3.098e-5**	0.31	0.099	**1.709e-3**
**cg21575225**	-0.10	0.020	6.428e-7	NA	NA	NA	NA	NA	NA

**cg06053307**	-0.33	0.064	7.562e-7	NA	NA	NA	NA	NA	NA
**cg17738628**	-0.22	0.044	8.896e-7	NA	NA	NA	NA	NA	NA
**cg06708913**	-0.24	0.047	8.901e-7	NA	NA	NA	NA	NA	NA
**cg13985437**	-0.15	0.029	9.061e-7	-0.07	0.029	**1.841e-2**	-0.11	0.038	**4.236e-3**
**cg21611682**	-0.13	0.026	9.195e-7	-0.09	0.020	**5.484e-6**	-0.12	0.028	**2.058e-5**
**cg13184736**	-0.31	0.062	9.433e-7	-0.28	0.062	**8.445e-6**	-0.28	0.065	**2.854e-5**
**cg11556164**	-0.19	0.038	1.029e-6	-0.19	0.038	**8.826e-7**	-0.07	0.074	3.270e-1
**cg09701682**	-0.18	0.037	1.030e-6	-0.03	0.026	1.423e-1	-0.09	0.044	**3.755e-2**
**cg00687135**	-0.20	0.040	1.035e-6	-0.11	0.037	**2.614e-3**	-0.03	0.046	4.696e-1
**cg02732789**	-0.27	0.053	1.093e-6	NA	NA	NA	NA	NA	NA
**cg26673040**	-0.28	0.055	1.124e-6	NA	NA	NA	NA	NA	NA
**cg26237233**	-0.12	0.024	1.304e-6	NA	NA	NA	NA	NA	NA
**cg00835193**	-0.45	0.090	1.666e-6	-0.14	0.041	**6.732e-4**	-0.19	0.051	**1.824e-4**
**cg01692968**	-0.28	0.057	1.668e-6	-0.15	0.033	**1.108e-5**	-0.22	0.053	b
**cg16611234**	-0.23	0.048	1.874e-6	-0.14	0.038	**2.204e-4**	-0.17	0.051	**7.150e-4**
**cg23079012**	-0.20	0.042	1.880e-6	NA	NA	NA	NA	NA	NA
**cg17651613**	-0.29	0.060	2.065e-6	NA	NA	NA	NA	NA	NA
**cg03450842**	-0.12	0.025	2.091e-6	-0.07	0.019	**2.613e-4**	-0.09	0.028	**9.140e-4**
**cg07339236**	-0.34	0.070	2.148e-6	-0.27	0.048	**3.548e-8**	-0.25	0.064	**1.669e-4**
**cg01278041**	-0.45	0.092	2.195e-6	-0.08	0.056	1.405e-1	-0.21	0.060	**4.862e-4**
**cg10532807**	-0.29	0.060	2.215e-6	-0.02	0.028	3.396e-1	0.007	0.047	8.702992e-1
**cg14316231**	-0.12	0.026	2.346e-6	-0.05	0.022	**1.282e-2**	-0.10	0.035	**2.211e-3**
**cg15021006**	-0.19	0.040	2.721e-6	NA	NA	NA	NA	NA	NA
**cg03547355**	-0.10	0.021	3.000e-6	-0.07	0.018	**4.857e-3**	-0.09	0.028	**2.047e-3**
**cg08709672**	-0.10	0.021	3.270e-6	-0.08	0.021	**9.848e-5**	-0.11	0.023	**3.666e-6**
**cg23068081**	-0.16	0.035	3.368e-6	NA	NA	NA	NA	NA	NA
**cg25120210**	-0.22	0.047	3.531e-6	0.04	0.035	2.486e-1	-0.05	0.048	3.036092e-1
**cg21901790**	-0.14	0.029	3.532e-6	NA	NA	NA	NA	NA	NA
**cg24843790**	-0.12	0.025	3.542e-6	NA	NA	NA	NA	NA	NA
**cg23238168**	-0.21	0.045	3.596e-6	NA	NA	NA	NA	NA	NA
**cg27215690**	-0.10	0.021	3.656e-6	NA	NA	NA	NA	NA	NA
**cg23161492**	-0.29	0.060	3.657e-6	-0.16	0.034	**3.106e-6**	-0.27	0.049	**2.020e-7**

**cg00971737**	-0.12	0.025	3.723e-6	-0.01	0.017	3.254e-1	-0.04	0.023	7.131e-2
**cg17287155**	-0.29	0.061	3.759e-6	-0.19	0.050	**1.583e-4**	-0.17	0.064	**6.695e-3**
**cg23110422**	-0.34	0.073	3.883e-6	-0.14	0.033	**3.784e-5**	-0.22	0.050	**1.657e-5**
**cg17625893**	-0.15	0.033	4.071e-6	NA	NA	NA	NA	NA	NA
**cg06679537**	0.21	0.045	4.104e-6	NA	NA	NA	NA	NA	NA
**cg00793186**	-0.17	0.036	4.288e-6	-0.05	0.029	6.403e-2	-0.07	0.038	**4.786e-2**
**cg25212025**	-0.16	0.035	4.523e-6	-0.10	0.032	**2.168e-3**	-0.11	0.038	**2.599e-3**
**cg18387338**	-0.20	0.042	4.538e-6	NA	NA	NA	NA	NA	NA
**cg00819417**	-0.21	0.046	4.644e-6	NA	NA	NA	NA	NA	NA
**cg09945032**	-0.14	0.030	4.6730e-6	NA	NA	NA	NA	NA	NA
**cg07776734**	-0.19	0.041	4.770e-6	NA	NA	NA	NA	NA	NA
**cg01260575**	-0.18	0.039	4.896e-6	NA	NA	NA	NA	NA	NA
**cg13383842**	-0.10	0.023	5.111e-6	NA	NA	NA	NA	NA	NA
**cg22287711**	-0.18	0.038	5.365e-6	NA	NA	NA	NA	NA	NA
**cg03384915**	-0.20	0.044	5.482e-6	-0.08	0.040	**3.369e-2**	-0.04	0.057	4.227e-1
**cg20174472**	-0.33	0.072	5.576e-6	NA	NA	NA	NA	NA	NA
**cg24361265**	-0.33	0.070	5.670e-6	-0.20	0.076	**9.476e-3**	-0.18	0.086	**3.970e-2**
**cg08774868**	0.26	0.056	5.734e-6	0.006	0.026	7.997e-1	0.04	0.037	1.878e-1
**cg24540678**	-0.21	0.046	5.826e-6	-0.13	0.031	**2.058e-5**	-0.10	0.045	**2.404e-2**
**cg05533761**	-0.23	0.050	5.958e-6	NA	NA	NA	NA	NA	NA
**cg24556382**	-0.27	0.059	6.294e-6	-0.07	0.034	**3.784e-2**	-0.11	0.047	**2.030e-2**
**cg01208318**	-0.25	0.055	6.888e-6	-0.06	0.045	1.611e-1	-0.04	0.068	5.131e-1
**cg07090714**	-0.11	0.023	6.974e-6	-0.04	0.050	4.261e-1	-0.14	0.028	**1.860e-6**

Supplementary Table 3 Regression coefficients (Coeff.), standard errors (SE), and nominal p-values for the top 20 CpGs in the analyses of UVR exposures and DNA methylation in the discover set. The analysis was adjusted for age, smoking, and time in freezer. The replicating CpGs are listed in Table 3, and are here marked in bold. CpG sites not present on the 450k chip (R_1 and R_2) are marked as NA (not applicable).

				Discovery set			Replication set R_1			Replication set R_2			Additional adjustment for cell type composition: Discovery set			Replication set R_1			Replication set R_2		
	Position	Gene	Relation	Ccoeff.	SE	$p_{nominal}$	Coeff.	SE	$p_{nominal}$	Coeff.	SE	$p_{nominal}$	Ccoeff.	SE	$p_{nominal}$	Coeff.	SE	$p_{nominal}$	Coeff.	SE	$p_{nominal}$
**Residential ambient UVR**																					
**cg08390979**	chr16:6094220	A2BP1	5'UTR	0.255	0.050	1.267e-6	0.043	0.027	0.1085	0.027	0.039	0.492	0.252	0.052	3.650e-06	0.04	0.026	0.135	0.013	0.037	0.721
**cg19163467**	chr10:120330711			0.069	0.013	1.567e-6	NA	NA	NA	NA	NA	NA	0.060	0.014	3.691e-05	NA	NA	NA	NA	NA	NA
**cg20744229**	chr11:66154113			0.219	0.045	2.901e-6	NA	NA	NA	NA	NA	NA	0.215	0.047	9.884e-06	NA	NA	NA	NA	NA	NA
**cg24788363**	chr2:105375363	LINC01114	TSS1500	0.081	0.017	3.807e-6	NA	NA	NA	NA	NA	NA	0.064	0.017	2.072e-04	NA	NA	NA	NA	NA	NA
**cg14885781**	chr20:34750927	EPB41L1	5'UTR	0.069	0.014	4.863e-6	NA	NA	NA	NA	NA	NA	0.056	0.014	1.039e-04	NA	NA	NA	NA	NA	NA
**cg14412016**	chr16:1434421	UNKL	Body	0.106	0.022	5.407e-6	NA	NA	NA	NA	NA	NA	0.094	0.019	1.757e-06	NA	NA	NA	NA	NA	NA
**cg09834582**	chr22:22780618			0.104	0.022	7.107e-6	NA	NA	NA	NA	NA	NA	0.082	0.019	3.164e-05	NA	NA	NA	NA	NA	NA
**cg10729739**	chr17:7835337	TRAPPC1	TSS200	0.070	0.015	7.404e-6	0.019	0.015	0.2023	-0.008	0.019	0.658	0.063	0.015	4.803e-05	0.02	0.015	0.131	-0.008	0.018	0.639
**cg09726829**	chr2:3651923	COLEC11	5'UTR	0.099	0.021	9.280e-6	NA	NA	NA	NA	NA	NA	0.090	0.022	6.203e-05	NA	NA	NA	NA	NA	NA
**cg10338668**	chr2:3649587	COLEC11	5'UTR	0.141	0.031	9.477e-6	NA	NA	NA	NA	NA	NA	0.121	0.029	6.695e-05	NA	NA	NA	NA	NA	NA
**cg18296707**	chr17:37824517	PNMT	Body	0.256	0.056	1.060e-5	NA	NA	NA	NA	NA	NA	0.245	0.058	4.348e-05	NA	NA	NA	NA	NA	NA

**cg04571819**	chr5:177645550	PHYKPL	Body	-0.117	0.026	1.395e-5	NA	NA	NA	NA	NA	NA	-0.105	0.025	7.710e-05	NA	NA	NA	NA	NA	NA
**cg02710875**	chr16:25226852	AQP8	TSS1500	0.073	0.016	1.484e-5	NA	NA	NA	NA	NA	NA	0.051	0.014	4.444e-04	NA	NA	NA	NA	NA	NA
**cg00367281**	chr8:42552470	CHRNB3	TSS200	0.080	0.018	2.130e-5	-0.023	0.014	0.0986	0.010	0.023	0.671	0.070	0.018	1.511e-04	-0.01	0.011	0.298	0.011	0.021	0.604
**cg27075774**	chr3:126943273			-0.205	0.047	2.259e-5	NA	NA	NA	NA	NA	NA	-0.177	0.044	8.741e-05	NA	NA	NA	NA	NA	NA
cg05860019	chr15:53095195			-0.132	0.030	2.279e-5	0.007	0.029	0.7997	-0.075	0.036	**0.043**	-0.119	0.031	1.774e-04	0.008	0.029	0.782	-0.071	0.036	0.051
**cg17068858**	chr15:80757855	ARNT2	Body	0.168	0.038	2.293e-5	NA	NA	NA	NA	NA	NA	0.111	0.028	1.154e-04	NA	NA	NA	NA	NA	NA
**cg26804446**	chr12:101050102			0.139	0.032	2.507e-5	NA	NA	NA	NA	NA	NA	0.130	0.033	1.390e-04	NA	NA	NA	NA	NA	NA
**cg02469944**	chr3:21666233	ZNF385D	Body	0.150	0.034	2.796e-5	NA	NA	NA	NA	NA	NA	0.114	0.033	6.575e-04	NA	NA	NA	NA	NA	NA
**cg13360325**	chr1:158054982	KIRREL	Body	0.135	0.031	2.823e-5	NA	NA	NA	NA	NA	NA	0.093	0.026	4.389e-04	NA	NA	NA	NA	NA	NA
**Lifetime no. of sunburns**																					
**cg19728199**	chr10:83989646	NRG3-AS1	Body	0.065	0.014	1.668e-5	NA	NA	NA	NA	NA	NA	0.066	0.014	8.590e-06	NA	NA	NA	NA	NA	NA
**cg07139440**	chr1:151030981	CDC42SE1	5'UTR	0.077	0.017	2.500e-5	-0.008	0.020	0.6969	0.034	0.03	0.269	0.066	0.016	6.660e-05	0.01	0.01	0.581	0.03	0.03	0.214
**cg09961264**	chr3:194818128	XXYLT1	Body	0.129	0.030	3.393e-5	NA	NA	NA	NA	NA	NA	0.105	0.024	3.594e-05	NA	NA	NA	NA	NA	NA
**cg13807035**	chr1:11774420	C1orf187	Body	0.084	0.019	3.539e-5	0.001	0.010	0.9261	0.007	0.020	0.725	0.075	0.018	7.793e-05	0.008	0.009	0.329	0.008	0.01	0.634

**cg08619295**	chr10:6364079			-0.057	0.013	3.695e-5	-0.001	0.009	0.8266	0.007	0.013	0.608	-0.049	0.012	6.282e-05	-0.001	0.007	0.883	0.005	0.01	0.680
**cg12006217**	chr14:69865914	ERH	TSS1500	0.078	0.018	3.716e-5	NA	NA	NA	NA	NA	NA	0.076	0.017	2.938e-05	NA	NA	NA	NA	NA	NA
**cg07970752**	chr16:817463	MSLN	Body	0.193	0.046	4.539e-5	-0.013	0.058	0.8158	0.095	0.076	0.210	0.194	0.046	5.593e-05	-0.02	0.05	0.611	0.11	0.07	0.157
**cg00033666**	chr15:96887043			0.069	0.016	4.696e-5	0.034	0.015	**0.0276**	0.009	0.023	0.702	0.067	0.015	3.870e-05	0.03	0.01	0.013	0.01		0.641
**cg27241845**	chr2:233250370			0.065	0.015	4.768e-5	-0.002	0.013	0.8643	0.028	0.018	0.120	0.059	0.015	2.179e-04	0.001	0.01	0.880	0.03	0.01	0.015
**cg06837596**	chr14:47509965	MDGA2	Body	0.084	0.020	5.292e-5	NA	NA	NA	NA	NA	NA	0.070	0.016	5.554e-05	NA	NA	NA	NA	NA	NA
**cg23517148**	chr19:5695366	LONP1	Body	0.069	0.016	5.631e-5	NA	NA	NA	NA	NA	NA	0.063	0.016	1.302e-04	NA	NA	NA	NA	NA	NA
**cg19218438**	chr22:49076091	FAM19A5	Body	0.416	0.100	5.925e-5	NA	NA	NA	NA	NA	NA	0.416	0.1043	1.002e-04	NA	NA	NA	NA	NA	NA
**cg18249107**	chr10:134996319	KNDC1	Body	-0.127	0.031	7.199e-5	0.029	0.017	0.0980	-0.002	0.025	0.931	-0.119	0.031	1.748e-04	0.03	0.01	0.049	0.007	0.02	0.764
**cg23238147**	chr1:15439013	KIAA1026	Body	0.055	0.013	7.279e-5	-0.018	0.012	0.1437	0.005	0.020	0.780	0.043	0.011	2.885e-04	-0.005	0.01	0.611	0.008	0.01	0.651
**cg05685892**	chr6:30421091			0.186	0.046	7.795e-5	0.027	0.028	0.3483	0.019	0.034	0.572	0.191	0.046	6.322e-05	0.03	0.02	0.199	0.02	0.03	0.390
**cg11669003**	chr22:46813946	CELSR1	Body	0.054	0.013	7.810e-5	NA	NA	NA	NA	NA	NA	0.046	0.011	7.950e-05	NA	NA	NA	NA	NA	NA
**cg21084508**	chr2:219905564	CCDC108	5'UTR	-0.095	0.023	8.982e-5	-0.008	0.016	0.6185	-0.027	0.028	0.340	-0.092	0.024	2.294e-04	-0.01	0.01	0.497	-0.02	0.02	0.342
**cg03772909**	chr3:138725311	PRR23A	TSS1500	-0.112	0.028	9.117e-5	-0.02	0.032	0.3739	-0.017	0.029	0.555	-0.110	0.027	1.177e-04	-0.03	0.03	0.354	-0.02	0.03	0.405

**cg06255943**	chr7:16922326	AGR3	TSS1500	0.076	0.019	1.054e-4	0.010	0.019	0.5777	0.018	0.038	0.630	0.069	0.017	9.919e-05	0.02	0.01	0.208	0.03	0.03	0.275
**cg10018845**	chr1:100232024	FRRS1	TSS1500	0.044	0.011	1.076e-4	0.0084	0.022	0.7109	0.005	0.036	0.889	0.036	0.009	3.069e-04	0.01	0.02	0.556	0.003	0.03	0.911
**Lifetime no. of sunbathing vacations**																					
**cg01884057**	chr2:25150051			0.161	0.035	9.736e-6	0.003	0.029	0.8982	0.005	0.043	0.906	0.151	0.033	1.136e-05	-0.009	0.02	0.741	0.033	0.04	0.431
**cg03795071**	chr8:47318907			-0.200	0.045	2.082e-5	-0.009	0.022	0.6728	0.033	0.026	0.208	-0.196	0.046	4.319e-05	-0.004	0.02	0.846	0.017	0.02	0.532
**cg02048220**	chr14:91141833	TTC7B	Body	-0.121	0.028	2.808e-5	-0.001	0.016	0.9522	-0.017	0.029	0.542	-0.101	0.026	1.989e-04	-0.012	0.01	0.373	-0.026	0.02	0.309
**cg08966326**	chr2:133012182	NCRNA00164	Body	-0.052	0.012	3.518e-5	-0.014	0.012	0.2543	0.0007	0.021	0.973	-0.051	0.012	6.844e-05	-0.011	0.01	0.320	-0.017	0.02	0.405
**cg17927223**	chr19:3750813	APBA3	3'UTR	-0.087	0.020	3.809e-5	-0.006	0.012	0.6307	0.006	0.021	0.751	-0.085	0.021	8.188e-05	-0.007	0.01	0.521	-0.004	0.02	0.848
**cg22711009**	chr10:118576132			-0.081	0.019	4.469e-5	-0.002	0.026	0.9208	-0.129	0.104	0.217	-0.082	0.019	4.580e-05	-0.001	0.02	0.956	-0.166	0.10	0.124
**cg27226098**	chr12:3751048	CRACR2A	Body	-0.108	0.026	5.723e-5	NA	NA	NA	NA	NA	NA	-0.110	0.025	3.053e-05	NA	NA	NA	NA	NA	NA
**cg14237287**	chr19:40768217	AKT2	Body	-0.106	0.025	5.745e-5	NA	NA	NA	NA	NA	NA	-0.110	0.025	3.069e-05	NA	NA	NA	NA	NA	NA
**cg04003327**	chr2:239007828	ESPNL	TSS1500	-0.260	0.063	6.140e-5	0.047	0.037	0.2007	0.006	0.053	0.910	-0.233	0.062	2.500e-04	0.040	0.03	0.270	-0.010	0.05	0.847
**cg18984282**	chr2:134326172	NCKAP5	TSS200	-0.148	0.036	6.841e-5	-0.018	0.025	0.4682	0.094	0.043	**0.031**	-0.150	0.037	8.116e-05	-0.018	0.02	0.467	0.089	0.04	0.048
**cg00776364**	chr16:28878700	SH2B1	Body	-0.060	0.014	7.289e-5	-0.019	0.025	0.4397	0.030	0.042	0.474	-0.057	0.014	7.688e-05	-0.006	0.02	0.778	0.007	0.03	0.844

**cg00641437**	chr3:138763875	PRR23C	TSS200	-0.127	0.031	7.964e-5	NA	NA	NA	NA	NA	NA	-0.120	0.030	1.120e-04	NA	NA	NA	NA	NA	NA
**cg21759745**	chr17:28672726			-0.084	0.020	8.605e-5	NA	NA	NA	NA	NA	NA	-0.083	0.020	6.897e-05	NA	NA	NA	NA	NA	NA
**cg13678706**	chr15:64439846			-0.099	0.024	8.968e-5	NA	NA	NA	NA	NA	NA	-0.089	0.024	3.029e-04	NA	NA	NA	NA	NA	NA
**cg00346376**	chr5:1110089	SLC12A7	Body	0.073	0.018	9.385e-5	-0.001	0.010	0.8491	0.014	0.015	0.355	0.070	0.017	8.454e-05	-0.001	0.008	0.873	0.008	0.01	0.607
**cg11747022**	chr19:2813926			-0.084	0.021	9.904e-5	-0.009	0.018	0.5994	0.005	0.022	0.798	-0.085	0.019	3.221e-05	-0.004	0.01	0.818	-0.005	0.02	0.805
**cg05580441**	chr17:78878844	RPTOR	Body	-0.090	0.022	1.019e-4	-0.001	0.010	0.8624	-0.012	0.023	0.600	-0.094	0.022	5.671e-05	-0.002	0.009	0.799	-0.015	0.02	0.506
**cg00316420**	chr17:2593957	KIAA0664	Body	-0.111	0.027	1.025e-4	0.008	0.013	0.5448	-0.019	0.023	0.408	-0.103	0.028	3.476e-04	0.004	0.01	0.696	-0.023	0.02	0.315
**cg23244421**	chr17:80056046	FASN	1stExon	0.050	0.012	1.057e-4	0.003	0.029	0.9010	0.016	0.040	0.687	0.052	0.012	4.212e-05	-0.006	0.02	0.805	0.051	0.03	0.147
**cg14335158**	chr2:64832404			-0.083	0.021	1.070e-4	NA	NA	NA	NA	NA	NA	-0.084	0.020	7.402e-05	NA	NA	NA	NA	NA	NA
**Lifetime no. of indoor tanning sessions**																					
**cg09928693**	chr10:1343202	ADARB2	Body	-0.253	0.047	2.999e-7	NA	NA	NA	NA	NA	NA	-0.211	0.045	6.036e-06	NA	NA	NA	NA	NA	NA
**cg22536580**	chr11:15095822	CALCB	5'UTR	0.190	0.040	4.263e-6	-0.012	0.023	0.5826	-0.003	0.027	0.901	0.186	0.041	1.511e-05	-0.011	0.023	0.623	-0.012	0.027	0.644
**cg15408237**	chr11:91974310			0.143	0.030	5.739e-6	-0.007	0.022	0.7498	-0.022	0.027	0.400	0.150	0.030	2.133e-06	-0.005	0.022	0.791	0.004	0.026	0.862
**cg17160384**	chr5:68462743	CCNB1	TSS200	0.068	0.014	6.224e-6	NA	NA	NA	NA	NA	NA	0.058	0.014	8.970e-05	NA	NA	NA	NA	NA	NA

**cg23022138**	chr10:1339887	ADARB2	Body	-0.169	0.036	7.232e-6	NA	NA	NA	NA	NA	NA	-0.158	0.038	5.060e-05	NA	NA	NA	NA	NA	NA
**cg25492350**	chr7:5280248			0.091	0.019	7.383e-6	-0.0005	0.018	0.9752	0.021	0.031	0.494	0.073	0.018	7.465e-05	0.004	0.016	0.798	0.0005	0.028	0.984
**cg22044398**	chr6:106583179			-0.087	0.018	7.615e-6	-0.005	0.010	0.5610	-0.004	0.012	0.747	-0.063	0.016	1.778e-04	-0.004	0.007	0.534	0.005	0.011	0.626
**cg10668195**	chr7:54768889			-0.133	0.028	8.397e-6	0.012	0.014	0.3809	-0.027	0.021	0.212	-0.096	0.023	7.053e-05	0.012	0.009	0.221	-0.014	0.019	0.459
**cg06138439**	chr16:54973128			0.046	0.010	9.644e-6	-0.010	0.007	0.1470	0.006	0.014	0.625	0.04	0.010	1.174e-04	-0.008	0.006	0.242	0.004	0.014	0.741
**cg25792367**	chr7:127291747	SND1	TSS1500	0.069	0.015	1.499e-5	-0.021	0.010	0.0330	0.010	0.016	0.547	0.06	0.015	1.380e-04	-0.020	0.009	0.042	0.007	0.016	0.656
**cg04028604**	chr15:69366848	MIR548H4	Body	0.070	0.016	2.154e-5	NA	NA	NA	NA	NA	NA	0.0593	0.015	1.182e-04	NA	NA	NA	NA	NA	NA
**cg09849302**	chr21:38378433	RIPPLY3	TSS1500	0.072	0.016	2.254e-5	NA	NA	NA	NA	NA	NA	0.054	0.014	1.962e-04	NA	NA	NA	NA	NA	NA
**cg14987787**	chr12:115105220			0.096	0.022	2.334e-5	-0.007	0.026	0.7836	0.012	0.039	0.756	0.074	0.020	3.864e-04	-0.008	0.024	0.740	0.009	0.038	0.804
**cg05043557**	chr17:6460360	PITPNM3	TSS1500	0.051	0.011	2.338e-5	0.011	0.012	0.3599	-0.009	0.014	0.508	0.053	0.012	1.130e-05	0.018	0.011	0.121	-0.006	0.015	0.669
**cg08762534**	chr14:51411787	PYGL	TSS1500	0.209	0.048	2.408e-5	NA	NA	NA	NA	NA	NA	0.184	0.046	1.242e-04	NA	NA	NA	NA	NA	NA
**cg06654103**	chr12:4920613	KCNA6	1stExon	0.088	0.020	2.475e-5	-0.002	0.008	0.8032	0.019	0.014	0.201	0.0885	0.020	2.227e-05	-0.0005	0.008	0.944	0.020	0.015	0.183
**cg18866599**	chr2:208635723			0.054	0.012	2.484e-5	0.072	0.038	0.0625	-0.046	0.058	0.422	0.047	0.011	9.542e-05	0.070	0.034	0.040	-0.046	0.056	0.418
**cg16655445**	chr6:105073697			-0.061	0.014	2.544e-5	NA	NA	NA	NA	NA	NA	-0.041	0.011	7.234e-04	NA	NA	NA	NA	NA	NA

**cg10242763**	chr7:82073690	CACNA2D1	TSS1500	0.060	0.014	2.792e-5	-0.005	0.013	0.6813	0.012	0.020	0.545	0.055	0.013	7.848e-05	0.0003	0.011	0.974	0.008	0.019	0.672
**cg13717880**	chr13:45563580	KIAA1704	TSS200	0.053	0.012	3.089e-5	-0.008	0.017	0.6513	0.014	0.023	0.548	0.045	0.012	2.066e-04	-0.015	0.016	0.342	0.004	0.020	0.811
**Cumulative UVR**																					
**cg01884057**	chr2:25150051			0.120	0.020	3.960e-8	-0.008	0.017	0.6397	0.019	0.0237	0.420	0.109	0.020	2.172e-07	-0.010	0.017	0.544	0.024	0.023	0.295
**cg09928693**	chr10:1343202	ADARB2	Body	-0.167	0.033	1.083e-6	NA	NA	NA	NA	NA	NA	-0.139	0.031	1.476e-05	NA	NA	NA	NA	NA	NA
**cg12928754**	chr7:30626557			-0.065	0.013	2.366e-6	NA	NA	NA	NA	NA	NA	-0.059	0.012	7.012e-06	NA	NA	NA	NA	NA	NA
**cg10589447**	chr12:115890128			0.030	0.006	2.960e-6	0.002	0.006	0.7630	0.001	0.006	0.777	0.025	0.005	3.977e-05	0.0005	0.006	0.929	0.002	0.006	0.707
**cg20667664**	chr17:38254448	NR1D1	Body	-0.065	0.013	3.119e-6	0.006	0.008	0.4316	-0.007	0.014	0.626	-0.054	0.013	8.481e-05	0.008	0.007	0.208	-0.0007	0.012	0.949
**cg18973515**	chr15:65713476	IGDCC4	Body	0.045	0.009	4.630e-6	0.005	0.008	0.4900	0.001	0.011	0.867	0.039	0.008	1.706e-05	0.004	0.007	0.557	0.008	0.009	0.376
**cg15277473**	chr15:70540396			-0.097	0.020	5.084e-6	NA	NA	NA	NA	NA	NA	-0.068	0.016	4.480e-05	NA	NA	NA	NA	NA	NA
**cg02343648**	chr10:102133165	C10orf75	TSS200	0.037	0.008	6.445e-6	-0.004	0.014	0.7473	-0.016	0.021	0.443	0.034	0.008	3.853e-05	-0.006	0.014	0.673	-0.022	0.022	0.321
**cg15423357**	chr2:25149977			0.136	0.029	6.572e-6	0.021	0.029	0.4818	0.024	0.040	0.550	0.111	0.027	8.942e-05	0.009	0.028	0.740	0.022	0.038	0.552
**cg25792367**	chr7:127291747	SND1	TSS1500	0.048	0.010	9.645e-6	-0.010	0.007	0.1713	-0.002	0.012	0.836	0.043	0.010	7.369e-05	-0.010	0.007	0.147	0.0003	0.011	0.973
**cg19500261**	chr20:4917818	SLC23A2	5'UTR	-0.067	0.014	1.133e-5	NA	NA	NA	NA	NA	NA	-0.061	0.014	5.270e-05	NA	NA	NA	NA	NA	NA

**cg13807496**	chr11:44332540	ALX4	TSS1500	0.039	0.008	1.179e-5	0.009	0.006	0.1723	-0.014	0.011	0.197	0.032	0.008	1.298e-04	0.007	0.006	0.241	-0.010	0.010	0.288
**cg06470626**	chr16:22364961	CDR2	Body	0.058	0.013	1.394e-5	0.003	0.009	0.6682	-0.020	0.015	0.203	0.038	0.010	4.054e-04	0.003	0.008	0.682	-0.003	0.013	0.779
**cg26738080**	chr3:52487733	TNNC1	Body	-0.053	0.011	1.444e-5	-0.008	0.008	0.3052	-0.009	0.013	0.482	-0.039	0.011	5.058e-04	-0.006	0.006	0.366	-0.006	0.013	0.644
**cg13157774**	chr17:4445291	MYBBP1A	Body	-0.058	0.013	1.503e-5	NA	NA	NA	NA	NA	NA	-0.055	0.012	2.349e-05	NA	NA	NA	NA	NA	NA
**cg10417571**	chr4:146851153	ZNF827	Body	-0.081	0.018	1.541e-5	NA	NA	NA	NA	NA	NA	-0.065	0.016	8.635e-05	NA	NA	NA	NA	NA	NA
**cg21875423**	chr2:217559867	IGFBP5	5'UTR	0.084	0.018	1.647e-5	-0.0005	0.012	0.9637	0.0210	0.020	0.302	0.067	0.018	3.685e-04	-0.005	0.011	0.600	0.036	0.018	0.057
**cg14174320**	chr11:66630151	PC	Body	-0.031	0.007	1.730e-5	-0.004	0.005	0.4751	-0.004	0.008	0.616	-0.022	0.006	7.492e-04	-0.001	0.003	0.710	-0.005	0.008	0.551
**cg15440524**	chr3:177534482	KCCAT211	TSS200	-0.056	0.012	1.806e-5	NA	NA	NA	NA	NA	NA	-0.047	0.011	7.302e-05	NA	NA	NA	NA	NA	NA
**cg11832956**	chr15:98196077			0.060	0.013	1.931e-5	0.013	0.012	0.2628	-0.020	0.015	0.166	0.062	0.014	2.225e-05	0.011	0.011	0.356	-0.014	0.015	0.342

Supplementary Table 4 Regression coefficients (Coeff.), standard errors (SE) and nominal p-values for the top 20 CpGs for each UVR exposure that replicated in R_1 or R_2 after adjustment for age, smoking, time in freezer, and hair color. Replicated p-values are shown in bold.

					Discovery			Replication set R1			Replication set R2		
**Exposure**	CpG	Pos	Gene	Relation	Coeff.	SE	$p_{nominal}$	Coeff.	SE	$p_{nominal}$	Coeff	SE	$p_{nominal}$
**Lifetime no. of sunburns**	cg00033666	chr15:96887043		Shore	0.07	0.01	9.44e-6	0.03	0.01	**0.019**	-0.01	0.024	0.667
**Lifetime no. of sunbathing vacations**	cg19577365	chr14:23574175		OpenSea	-0.09	0.02	4.37e-5	0.01	0.01	0.198	0.04	0.020	**0.015**

Supplementary Table 5 Regression coefficients (Coeff.), standard errors (SE), and nominal p-values in all three data sets for the association between mean methylation and the UVR exposures. Adjusted for age, smoking, and time in freezer.

	Discovery set			Replication set R1			Replication set R2		
	Coeff.	SE	$p_{nominal}$	Coeff.	SE	$p_{nominal}$	Coeff.	SE	$p_{nominal}$
**Residential ambient UVR**	0.0005	0.0002	0.06	-0.0004	0.0003	0.18	-0.0001	0.0003	0.69
**Lifetime no. of sunburns**	0.0003	0.0002	0.13	-0.0003	0.0002	0.21	-0.0003	0.0003	0.29
**Lifetime no. of sunbathing vacations**	1.924e-5	0.0002	0.93	1.15e-4	0.0002	0.68	8.10e-5	0.0003	0.81
**Lifetime no. of indoor tanning sessions**	-0.0001	0.0002	0.44	-0.0002	0.0002	0.23	-0.0002	0.0002	0.41
**Cumulative UVR[a]**	-7.55e-5	0.0001	0.61	-1.09e-4	0.0001	0.53	-9.33e-5	0.0002	0.65

[a]Sunbathing vacations and indoor tanning

Supplementary Table 6 Regression analysis of LINE-1 sub-families in the discovery set using a linear mixed model, where the CpGs are clustered into their respective sub-families. The DNA methylation was modelled as the outcome, UVR as the exposure and adjusted for age, smoking, and time in freezer. Each CpG position within a sub-family had a random effect. We present regression coefficients (Coeff.) and nominal p-values for the overall association between CpGs in a LINE-1 subfamily for each UVR exposure.

	Residential ambient UVR		Lifetime no. of sunburns		Lifetime no. of sunbathing vacations		Lifetime no. of indoor tanning sessions		Cumulative UVR[a]	
**LINE-1 sub-family**	Coeff.	$p_{nominal}$	Coeff.	$p_{nominal}$	Coeff.	$p_{nominal}$	Coeff.	$p_{nominal}$	Coeff.	$p_{nominal}$
**L1PA12**	-1.04e-2	0.11	0.007	0.11	-1.42e-3	0.80	4.12e-3	0.41	0.001	0.59
**L1PBb**	2.86e-2	0.12	-0.003	0.81	-1.82e-2	0.26	-9.19e-3	0.52	-0.009	0.31
**L1M2**	-1.09e-2	0.12	0.001	0.82	-4.18e-3	0.50	7.15e-3	0.19	0.002	0.51
**L1MC5**	7.99e-3	0.14	0.002	0.45	-3.70e-3	0.43	-4.36e-3	0.29	-0.003	0.18
**L1HS**	-9.72e-3	0.17	-0.001	0.75	-5.14e-3	0.41	-1.06e-3	0.84	-0.003	0.41
**L1PA2**	-8.44e-3	0.18	-0.0007	0.86	-6.78e-3	0.21	-1.15e-3	0.81	-0.003	0.29
**L1PA13**	8.02e-3	0.20	0.002	0.54	-2.11e-3	0.70	-3.51e-3	0.46	-0.002	0.41
**L1PA17**	9.14e-3	0.20	-0.0007	0.895	-4.73e-3	0.45	3.24e-3	0.55	-0.0004	0.89
**L1M3c**	1.32e-2	0.22	0.006	0.44	-6.33e-3	0.50	-4.58e-3	0.58	-0.006	0.23
**L1ME2z**	6.28e-3	0.23	0.003	0.40	-5.94e-3	0.20	-3.34e-3	0.40	-0.004	0.13
**L1MEg**	7.33e-3	0.24	0.003	0.43	-3.08e-3	0.57	-5.59e-3	0.24	-0.004	0.17
**L1MEc**	7.10e-3	0.24	0.0004	0.92	-3.10e-3	0.56	-6.00e-3	0.19	-0.004	0.16
**L1P4d**	1.14e-2	0.25	-0.002	0.77	-4.37e-3	0.61	-8.07e-3	0.28	-0.006	0.19
**L1MEd**	7.05e-3	0.26	0.002	0.51	-1.75e-3	0.75	-6.12e-3	0.20	-0.004	0.21
**L1MC2**	6.80e-3	0.28	0.002	0.60	-2.92e-3	0.60	-2.08e-3	0.66	-0.002	0.49
**L1ME3A**	5.73e-3	0.30	0.002	0.49	-4.91e-3	0.31	-3.10e-3	0.46	-0.003	0.18
**L1PA16**	5.27e-3	0.30	-0.0009	0.80	-2.82e-3	0.52	-7.79e-4	0.84	-0.001	0.52
**L1PB3**	8.53e-3	0.31	-0.005	0.39	4.64e-3	0.53	2.37e-3	0.71	0.003	0.49
**L1PA3**	-7.03e-3	0.32	0.0002	0.96	-7.20e-3	0.24	-2.56e-3	0.63	-0.004	0.26
**L1M5**	5.24e-3	0.33	0.003	0.37	-4.59e-3	0.33	-3.52e-3	0.39	-0.003	0.20
**L1M2c**	7.67e-3	0.35	0.005	0.37	-7.71e-3	0.28	-1.16e-2	0.06	-0.009	0.03
**L1PA8A**	9.22e-3	0.36	-0.001	0.79	-4.88e-3	0.57	-8.79e-4	0.90	-0.003	0.54
**L1M3f**	7.10e-3	0.37	-0.002	0.62	-6.23e-3	0.37	8.03e-3	0.18	0.0006	0.86
**L1MB1**	6.11e-3	0.38	0.0005	0.90	-2.09e-3	0.73	-2.32e-3	0.66	-0.002	0.52
**L1MCb**	7.06e-3	0.39	-0.002	0.71	-2.82e-3	0.69	-6.51e-3	0.30	-0.004	0.33

**L1MEa**	6.85e-3	0.39	0.001	0.85	-7.38e-3	0.30	-4.75e-3	0.44	-0.004	0.26
**L1PB2**	5.81e-3	0.40	0.004	0.35	-8.58e-3	0.15	-1.72e-3	0.74	-0.004	0.23
**L1P4**	6.11e-3	0.41	0.003	0.57	-4.03e-3	0.53	1.15e-4	0.98	-0.001	0.64
**L1PA14**	5.22e-3	0.42	0.002	0.56	-6.00e-3	0.29	-3.66e-3	0.46	-0.003	0.25
**L1P1**	9.68e-3	0.42	-0.005	0.51	3.89e-3	0.70	-8.96e-5	0.99	0.001	0.76
**L1ME2**	4.52e-3	0.43	0.003	0.43	-4.15e-3	0.41	-4.31e-3	0.32	-0.004	0.16
**L1MA4**	4.87e-3	0.43	0.002	0.57	-2.17e-3	0.69	-4.004e-3	0.40	-0.003	0.29
**L1PA10**	4.70e-3	0.44	0.001	0.66	-2.27e-3	0.67	-2.55e-3	0.58	-0.002	0.41
**L1MA4A**	5.41e-3	0.44	0.003	0.53	-3.85e-3	0.52	-2.41e-3	0.65	-0.002	0.46
**HAL1b**	4.78e-3	0.44	0.002	0.55	-4.83e-3	0.38	-2.67e-3	0.57	-0.003	0.27
**L1MA5A**	-9.18e-3	0.44	-0.009	0.30	1.31e-4	0.98	-2.38e-4	0.97	0.001	0.82
**L1ME3C**	4.24e-3	0.45	0.0018	0.64	-2.00e-3	0.68	2.86e-4	0.94	-0.0004	0.87
**L1ME4a**	3.85e-3	0.45	0.003	0.30	-3.85e-3	0.39	-2.55e-3	0.51	-0.002	0.29
**L1PA15**	5.01e-3	0.46	0.002	0.57	-2.26e-3	0.70	-3.95e-3	0.45	-0.003	0.36
**L1MCc**	7.41e-3	0.47	0.002	0.77	-4.44e-3	0.61	-1.01e-2	0.19	-0.007	0.16
**L1ME5**	4.27e-3	0.47	0.003	0.44	-9.90e-4	0.84	-4.62e-3	0.30	-0.002	0.35
**L1P4c**	8.88e-3	0.48	-0.003	0.68	-6.33e-3	0.56	-1.07e-2	0.26	-0.009	0.15
**L1ME3E**	4.00e-3	0.50	0.003	0.37	-2.62e-3	0.61	-3.63e-3	0.42	-0.002	0.34
**L1MC**	3.46e-3	0.50	0.001	0.61	-5.10e-3	0.25	-1.73e-3	0.66	-0.003	0.23
**L1MB4**	3.91e-3	0.50	0.003	0.41	-4.39e-3	0.39	-1.49e-3	0.73	-0.002	0.39
**L1ME1**	3.96e-3	0.51	0.001	0.77	-4.60e-3	0.38	-3.64e-3	0.42	-0.003	0.22
**HAL1**	3.87e-3	0.52	0.001	0.69	-3.85e-3	0.46	-3.85e-3	0.40	-0.003	0.23
**L1ME3D**	3.77e-3	0.52	0.002	0.57	-5.13e-3	0.32	-5.59e-3	0.21	-0.004	0.12
**L1PREC2**	4.61e-3	0.53	-0.001	0.82	-3.35e-4	0.95	-2.64e-3	0.63	-0.001	0.62
**L1MB5**	3.85e-3	0.53	0.002	0.58	-3.76e-3	0.48	-2.61e-3	0.57	-0.003	0.33
**L1M4b**	3.98e-3	0.53	0.006	0.14	-5.40e-3	0.33	-2.96e-3	0.54	-0.003	0.25
**L1M**	5.18e-3	0.55	0.001	0.85	-7.05e-3	0.34	1.85e-3	0.77	-0.002	0.62
**L1MA5**	3.68e-3	0.57	0.001	0.71	-2.40e-3	0.67	-2.87e-3	0.56	-0.001	0.61
**L1MD2**	3.47e-3	0.57	0.004	0.35	-4.69e-3	0.38	-4.35e-3	0.35	-0.004	0.16
**L1MC1**	3.51e-3	0.58	0.0007	0.86	-4.69e-3	0.41	-4.22e-3	0.39	-0.004	0.20
**L1PA8**	3.51e-3	0.59	0.002	0.62	-4.05e-3	0.48	-4.93e-3	0.32	-0.004	0.19
**L1MD1**	3.16e-3	0.61	-0.0006	0.87	-5.97e-3	0.27	-4.46e-3	0.34	-0.004	0.13

**L1MB3**	2.95e-3	0.61	0.001	0.65	-3.99e-3	0.43	-2.68e-3	0.54	-0.003	0.27
**L1MEf**	3.14e-3	0.63	0.001	0.78	-6.86e-3	0.22	-5.59e-3	0.25	-0.006	0.07
**L1M4**	2.69e-3	0.65	0.001	0.71	-5.62e-3	0.27	-3.87e-3	0.39	-0.004	0.16
**L1MC4a**	2.63e-3	0.65	0.004	0.29	-3.82e-3	0.45	-2.18e-3	0.62	-0.002	0.33
**L1MB2**	2.73e-3	0.65	0.002	0.57	-2.03e-3	0.70	-3.58e-3	0.43	-0.002	0.34
**L1PB4**	3.02e-3	0.65	0.0005	0.89	-4.03e-3	0.49	-4.57e-3	0.37	-0.004	0.25
**L1PBa1**	-3.29e-3	0.65	0.002	0.58	-3.25e-3	0.61	-5.25e-3	0.35	-0.004	0.25
**L1P2**	3.06e-3	0.67	0.002	0.58	1.17e-3	0.85	-3.18e-3	0.56	-0.001	0.70
**L1MC4**	2.48e-3	0.67	0.003	0.43	-5.04e-3	0.33	-4.14e-3	0.35	-0.004	0.17
**L1ME3F**	2.73e-3	0.68	0.002	0.66	-3.80e-3	0.51	5.75e-4	0.91	-0.001	0.73
**L1MC3**	2.43e-3	0.68	0.002	0.55	-2.31e-3	0.66	-2.26e-3	0.61	-0.002	0.48
**L1MD3**	2.44e-3	0.69	0.005	0.21	-3.81e-3	0.47	-4.68e-3	0.31	-0.004	0.18
**L1M2a**	3.70e-3	0.69	0.0001	0.98	-9.80e-3	0.23	-1.07e-2	0.13	-0.009	0.04
**L1MB7**	2.38e-3	0.70	0.003	0.43	-4.45e-3	0.41	-2.71e-3	0.56	-0.003	0.29
**L1PA6**	-2.19e-3	0.70	0.001	0.79	-1.73e-3	0.73	-2.77e-3	0.53	-0.002	0.46
**L1MEg1**	-3.63e-3	0.70	0.012	0.07	-6.17e-3	0.45	1.36e-2	0.05	0.004	0.41
**L1MA1**	2.54e-3	0.70	-0.002	0.65	-4.50e-3	0.44	-4.68e-3	0.36	-0.004	0.20
**L1M1**	2.13e-3	0.71	0.003	0.39	8.66e-4	0.86	-3.27e-3	0.45	-0.001	0.60
**L1MB8**	2.27e-3	0.71	0.0007	0.85	-4.75e-3	0.38	-3.78e-3	0.42	-0.003	0.22
**L1MDb**	-2.17e-3	0.72	0.002	0.61	2.16e-3	0.68	6.51e-3	0.15	0.004	0.14
**L1MDa**	2.19e-3	0.72	0.005	0.26	-8.62e-5	0.98	-3.76e-3	0.42	-0.002	0.49
**L1PBa**	2.34e-3	0.73	-0.001	0.72	-9.29e-3	0.11	5.81e-4	0.91	-0.003	0.38
**L1ME3B**	1.84e-3	0.73	0.003	0.47	-4.49e-3	0.35	-2.75e-3	0.50	-0.003	0.26
**L1PA7**	2.11e-3	0.74	0.0009	0.83	-3.87e-3	0.50	-2.57e-3	0.60	-0.003	0.35
**L1M6**	1.80e-3	0.75	0.003	0.42	-7.48e-3	0.13	-9.32e-4	0.83	-0.002	0.33
**L1PB1**	1.82e-3	0.77	0.003	0.55	-2.39e-3	0.66	-1.30e-3	0.78	-0.001	0.61
**L1PB**	-1.59e-3	0.80	0.006	0.20	-2.24e-3	0.69	-5.25e-4	0.91	-0.001	0.68
**L1MA2**	1.43e-3	0.81	0.0007	0.85	-4.24e-3	0.41	-4.65e-3	0.30	-0.004	0.19
**L1MEb**	-2.81e-3	0.81	-0.005	0.55	-1.15e-2	0.25	-7.29e-3	0.41	-0.008	0.17
**L1ME3**	1.40e-3	0.81	0.002	0.62	-3.04e-3	0.56	-4.31e-3	0.34	-0.003	0.28
**L1MA8**	1.45e-3	0.82	0.003	0.45	-3.82e-3	0.49	-4.92e-3	0.31	-0.004	0.20
**L1MA7**	-1.60e-3	0.82	0.003	0.49	-8.90e-3	0.15	1.32e-3	0.80	-0.002	0.48

**L1M4c**	1.36e-3	0.83	0.003	0.42	-3.28e-3	0.56	-5.48e-3	0.26	-0.004	0.19
**L1PA11**	1.27e-3	0.85	0.003	0.44	-1.39e-3	0.81	-1.74e-3	0.73	-0.002	0.51
**L1P3**	-1.29e-3	0.85	0.003	0.54	-4.00e-3	0.51	2.92e-3	0.58	0.0003	0.92
**L1MA6**	1.57e-3	0.87	0.004	0.59	-5.77e-3	0.51	-4.84e-3	0.53	-0.004	0.40
**L1PA4**	7.16e-4	0.91	0.003	0.47	-4.55e-3	0.42	-3.31e-3	0.49	-0.003	0.25
**L1MA3**	6.04e-4	0.92	-0.001	0.76	-3.84e-3	0.47	-2.85e-3	0.54	-0.003	0.31
**L1P5**	6.83e-4	0.9	0.003	0.59	1.68e-3	0.78	-3.09e-3	0.57	-0.001	0.66
**L1MD**	5.59e-4	0.92	-0.0009	0.82	-5.52e-3	0.30	-3.48e-3	0.45	-0.004	0.19
**L1M3e**	1.22e-3	0.94	-0.02	0.15	-5.35e-3	0.72	-7.18e-3	0.59	-0.005	0.55
**L1MA10**	5.35e-4	0.94	0.002	0.73	2.47e-3	0.72	3.46e-4	0.95	0.0009	0.81
**L1PA15-16**	4.71e-4	0.95	0.004	0.51	-9.03e-3	0.19	1.38e-3	0.82	-0.003	0.47
**L1M3**	-3.08e-4	0.96	0.005	0.29	-6.18e-3	0.25	-3.02e-4	0.94	-0.002	0.37
**L1MCa**	-3.60e-4	0.96	-0.001	0.83	-3.61e-3	0.58	1.20e-3	0.83	-0.001	0.62
**L1MA9**	-1.99e-4	0.97	0.005	0.27	-4.28e-3	0.42	-1.04e-3	0.82	-0.002	0.48
**L1PA5**	1.86e-4	0.97	0.003	0.49	-5.06e-3	0.34	-3.05e-3	0.51	-0.003	0.26
**L1M7**	7.61e-5	0.99	0.002	0.70	-4.99e-3	0.41	-3.27e-3	0.53	-0.003	0.30

[a]Sunbathing vacations and indoor tanning
